# Height and subjective body image are associated with suicide ideation among Korean adolescents

**DOI:** 10.3389/fpsyt.2023.1172940

**Published:** 2023-06-12

**Authors:** Kyungchul Song, Junghan Lee, San Lee, Soyoung Jeon, Hye Sun Lee, Ho-Seong Kim, Hyun Wook Chae

**Affiliations:** ^1^Department of Pediatrics, College of Medicine, Yonsei University, Seoul, Republic of Korea; ^2^Department of Psychiatry, Institute of Behavioral Sciences in Medicine, College of Medicine, Yonsei University, Seoul, Republic of Korea; ^3^Biostatistics Collaboration Unit, College of Medicine, Yonsei University, Seoul, Republic of Korea

**Keywords:** height, body image (MeSH), suicide, obesity, adolescent

## Abstract

**Introduction:**

Suicide is the leading cause of death among Korean adolescents. Suicide has been found to be associated with body mass index (BMI), height, and subjective body image among adults, but investigations of these associations among adolescents are limited. Thus, we aimed to examine to what extent suicide ideation is associated with height, BMI, and subjective body image among Korean adolescents.

**Methods:**

This study examined the data of 6,261 adolescents, selected from a nationally representative survey. The participants were divided into subgroups by sex, suicide ideation, and subjective body image. Logistic regression analyses were performed to examine the association of suicide ideation with height, BMI, and subjective body image.

**Results:**

The proportion of perceived obesity was high in the total sample; the height Z-score was lower for the group with suicide ideation than the group without suicide ideation; the height Z-scores were also lower for female participants with suicide ideation than those female participants without suicide ideation. The proportions of depressed mood, suicide ideation, and suicide attempts were higher among the total sample and female participants with perceived obesity than among those with a normal body image. On logistic regression, perceived obesity was positively associated with suicide ideation even after adjusting for age, height Z-score, weight Z-score, and depressed mood, whereas height Z-score was negatively associated with suicide ideation. These relationships were more prominent among female participants than among male participants.

**Conclusion:**

Low height and perceived obesity, not real obesity, are associated with suicide ideation among Korean adolescents. These findings indicate that the need for an integrated approach to growth, body image, and suicide in adolescents is warranted.

## 1. Introduction

According to the WHO, suicide is the third leading cause of death among adolescents globally (1). Moreover, the suicide rate in South Korea is the highest among the Organization for Economic Co-operation and Development countries, and suicide is the leading cause of death among Korean adolescents (1–3). In global studies, the prevalence of depression and suicide ideation among adolescents has been reported at 12.0 and 14.0%, respectively (4, 5). Among Korean adolescents, these rates increased to 13.6 and 19.1%, respectively (6, 7). Although it is impossible to prevent all suicides because of the multifactorial complexity, it is important to clarify the modifiable factors of suicide (8).

Short stature, that is, height below the third percentile or more than 2 standard deviations below the corresponding mean height for those of the same sex, age, and race (9), is associated with psychosocial problems and medical conditions, such as poor diet, genetic predisposition, physical activity, and underlying diseases (10, 11). In children and adolescents, regardless of cause, short stature leads to functional impairment in daily life (12). Youths with short stature tend to have behavioral problems as well as negative social experiences, including teasing, lower social acceptance, and lack of friends (13, 14). Although short stature often represents a normal variation in the general population, negative social stereotypes associated with short stature still exist, resulting in poor psychosocial performance in short children who are actually normal (15). A negative association between all-cause mortality and adult height in a cohort study implied that short stature was positively associated with mortality risk owing to genetics, socioeconomic factors, nutritional factors, or a previous infection marker in childhood (10, 11). In a Swedish study, short height was associated with an increased risk of suicide attempts among young men (11). A systematic review reported that depression might be related to relevant neurobiological dysfunctions, in particular, immune-inflammatory abnormalities (16). However, few studies in the literature have examined the exact association between height and suicide among adolescents.

Obesity is a complex disease associated with medical conditions, including chronic diseases as well as psychological distress (17, 18). Bjerkeset et al. (19) reported that body mass index (BMI) was positively associated with the risk of depression among adults (19). Moreover, a population-based study demonstrated that BMI was positively associated with suicide ideation among women (20). Given that the global prevalence of obesity among youth increased from 0.9 to 7.8% in male subjects and from 0.7 to 5.6% in female subjects from 1975 to 2016, the attention paid to the association between obesity and mental health has increased (18). Similar to obese adults, obese adolescents also appear to engage higher in suicide ideation (21). Despite the association between BMI and suicide ideation, it is still unclear if these aspects are directly affected by each other or not.

Obesity usually has a negative influence in the form of psychological distress on weight stigma (22). A meta-analysis reported that negative weight stigma is related to psychological distress, including depression and anxiety (23). Owing to negative attitudes, including prejudice and discrimination, which are widely prevalent in media, healthcare, and educational settings, obese individuals are vulnerable to psychological distress (22): negative attitudes typically stem from the social stereotype that obese people are lazy and unmotivated (24). Although weight stigma can motivate some people to lose weight, it can also worsen obesity by decreasing physical activity and increasing binge eating, owing to social isolation (24). Hunger et al. (25) reported that weight-based discrimination was positively related to suicide ideation among adults. Gavin et al. (26) showed that body image dissatisfaction as well as obesity might be risk factors for depression among women. Body image is strongly related to the self-esteem of young adults, and perceived poor body image is usually prevalent among women. Body image dissatisfaction is known to be associated with being overweight (27), and it can mediate the relationship between obesity and depression (26, 27). Moreover, body image is related to eating disorders. A population-based study reported that disordered eating behaviors are related to body image as well as body weight and BMI among adolescents (28). Calugi et al. (29) reported that the assessment of body image should be considered in the treatment of anorexia nervosa. While obesity, body image, and depression seem to be correlated, studies investigating the association between BMI and subjective body image with suicide ideation among adolescents are limited.

Despite several reviews of the associations among height, body weight, subjective body image, and suicidal ideation, it is still unclear which of these factors could be primary targets of suicide prevention. Therefore, this study aimed to investigate the associations between suicide ideation and BMI, height, and subjective body image among Korean adolescents using data from the Korea National Health and Nutrition Examination Survey (KNHANES). We aimed to test the following: (1) height, BMI, and body image are associated with psychological distress including suicidal ideation among adolescents and (2) negative body image might affect mental health even in adolescents without obesity.

## 2. Methods

### 2.1. Study population

**Figure 1:**
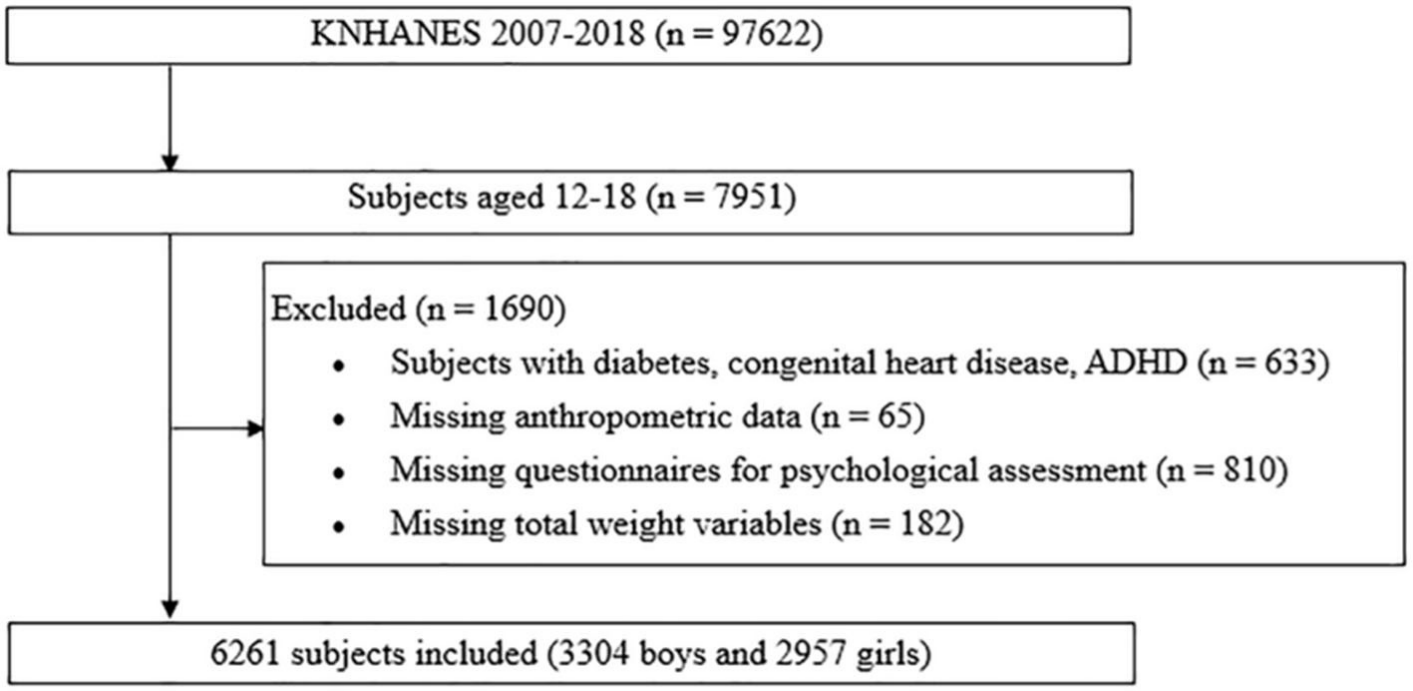
Design and flowchart of the study population. KNHANES, Korea National Health and Nutrition Examination Survey; ADHD, attention deficit hyperactivity syndrome.

We conducted a cross-sectional study of 6,261 adolescents aged 12–18 years included in four phases of the KNHANES: IV (2007–2009), V (2010–2012), VI (2013–2015), and VII (2016–2018). Figure 1 shows a flowchart for the study design and patient inclusion.

The KNHANES is a nationally representative cross-sectional survey, with a stratified and multistage sampling design, conducted by the Korea Centers for Disease Control and Prevention, based on the National Health Promotion Act (30). This cross-sectional survey involves a two-step stratified sampling method using sampling units and households as the primary and secondary sampling units, respectively. The sample weights for sample participants are constructed by accounting for the complex survey design, survey non-response, and post-stratification with a multistage and stratified systematic sampling design, considering non-responders to represent the Korean population. The data from health interviews, health examinations, and nutrition surveys provide information about health status and behavior, socioeconomic demographics, and laboratory tests. Trained interviewers and medical technicians conduct interviews using a structured questionnaire.

### 2.2. Data collection and study variables

Data on demographic, anthropometric, and behavioral characteristics were collected. Height and weight were measured using standard protocols, and BMI was calculated as weight (kg) divided by height squared (m^2^). Height and BMI were presented as Z-scores based on the 2017 Korean National Growth Charts (31). Short stature was defined as a height Z-score below −2.0.

Depressed mood, suicide ideation, suicide plans, and suicide attempts were assessed *via* participants' responses to the following questions (possible responses were “yes” and “no”): “During the past year, did you ever face a period of 2 weeks or more when you felt sad, blue, or depressed nearly every day?,” “During the past year, did you think about dying by suicide?,” “During the past year, did you seriously plan a suicide attempt(s)?,” and “Have you attempted suicide during the past year?” These questions are included in the WHO Composite International Diagnostic Interview-Short Form, which has been validated as a cost-effective screening method for public surveys and is a well-documented predictor of suicide attempts that have been previously used in other surveys (32, 33). Subjective body image was assessed by asking, “In your opinion, how do you perceive your body?” The possible responses included “lean,” “normal,” and “obese.”

### 2.3. Statistical analyses

The sampling weights were considered in all analyses to report representative estimates of the Korean population. The data were analyzed using SAS version 9.4 (SAS Inc., Cary, NC, USA). All continuous variables were expressed as weighted means with standard errors, whereas categorical variables are expressed as weighted percentages with standard errors. We divided the participants into subgroups according to sex, height Z-score, suicide ideation, subjective body image, and KNHANES phase. Independent two-sample *t*-test and analysis of variance were used to compare the mean values of continuous variables, and the Rao–Scott chi-square test was used to compare the weighted percentages of categorical variables. Logistic regression analyses were performed to explain the relationship between suicide ideation as a dependent variable and various markers. A *p*-value < 0.05 was considered statistically significant.

## 3. Results

### 3.1. Baseline characteristics of the participants according to sex

Among the participants, 9.55% reported depressed mood, 9.20% reported suicide ideation, 1.21% had a suicide plan, and 1.99% had attempted suicide (Table 1). Among male participants, 7.62% exhibited depressed mood, 6.42% exhibited suicide ideation, 0.76% had a suicide plan, and 1.50% had attempted suicide. Among female participants, 11.77% reported depressed mood, 12.40% reported suicide ideation, 1.69% had a suicide plan, and 2.47% had attempted suicide. The proportion of participants with obesity was higher among male participants than female participants, whereas the proportion of those with perceived obesity was higher among female participants (all *p* < 0.001). The proportion of the underweight and those with a lean body image was higher among male participants than in female participants (*p* < 0.001). In contrast, the proportions of those with depressed mood and suicide ideation were higher among female participants than in male participants (all *p* < 0.001).

**Table 1:** Baseline characteristics according to sex.

	**Total (*n =* 6,261)**	**Male (*n =* 3,304)**	**Female (*n* = 2,957)**	***p***
Age, y	15.11 (0.03)	15.11 (0.04)	15.11 (0.04)	0.974
Height Z-score	0.21 (0.02)	0.23 (0.02)	0.19 (0.02)	0.229
**Height group**				**0.389**
Z-score < −2	1.63 (0.19)	1.82 (0.28)	1.41 (0.25)	
−2 ≤ Z-score < 0	40.05 (0.77)	39.68 (1.04)	40.47 (1.04)	
0 ≤ Z-score < 2	54.14 (0.78)	53.96 (1.05)	54.35 (1.07)	
Z-score ≥ 2	4.18 (0.30)	4.54 (0.43)	3.77 (0.41)	
Weight Z-score	0.08 (0.02)	0.06 (0.03)	0.10 (0.03)	0.291
BMI Z-score	−0.05 (0.02)	−0.09 (0.03)	0.00 (0.03)	0.024
**BMI percentile**				<**0.001**
Underweight	8.98 (0.44)	10.66 (0.64)	7.06 (0.53)	
Normal	71.26 (0.66)	69.18 (0.94)	73.64 (0.95)	
Overweight	9.06 (0.41)	8.96 (0.56)	9.18 (0.59)	
Obesity	10.70 (0.48)	11.20 (0.67)	10.12 (0.68)	
**Subjective body image**				<**0.001**
Lean	26.00 (0.67)	34.62 (1.00)	16.10 (0.78)	
Normal	42.56 (0.72)	37.13 (0.98)	48.80 (1.10)	
Obese	31.44 (0.69)	28.25 (0.87)	35.10 (1.07)	
Depressed mood	9.55 (0.46)	7.62 (0.57)	11.77 (0.72)	< 0.001
Suicide ideation	9.20 (0.44)	6.42 (0.52)	12.40 (0.68)	< 0.001
Suicide plan	1.21 (0.24)	0.76 (0.29)	1.69 (0.40)	0.070
Suicide attempt	1.99 (0.29)	1.50 (0.38)	2.47 (0.43)	0.098

Continuous variables are presented as means (standard error) and categorical data as percentages (standard error). BMI, body mass index.

### 3.2. Characteristics of the participants according to suicide ideation

In the subgroup analysis, according to suicide ideation, the height Z-score was lower, and the proportions of individuals with perceived obesity, depressed mood, and a suicide plan and attempt were higher among the participants with suicide ideation than those without suicide ideation (*p* = 0.025 for height Z-score, all other *p* < 0.001) (Table 2). Among male participants, the proportions of individuals with depressed mood and a suicide plan and attempt were higher among those with suicide ideation than those without (all *p* < 0.001). For female participants, the height Z-scores were lower, and the proportions of individuals with short stature, perceived obesity, depressed mood, and suicide plan and attempt were higher in the group with suicide ideation than in the group without (*p* = 0.033 for height Z-score vs. *p* = 0.047 for short stature, all other *p* < 0.001).

**Table 2:** Comparison of subjects according to suicide ideation.

	**Total**			**Male**			**Female**		
	**Yes**	**No**	***P***	**Yes**	**No**	***P***	**Yes**	**No**	***P***
Age, y	15.207 (0.086)	15.097 (0.030)	0.223	15.262 (0.139)	15.098 (0.039)	0.254	15.175 (0.110)	15.095 (0.045)	0.505
Height Z-score	0.106 (0.051)	0.226 (0.018)	0.025	0.169 (0.075)	0.236 (0.025)	0.393	0.069 (0.066)	0.215 (0.024)	0.033
**Height group**			**0.208**			**0.967**			**0.047**
Z-score < -2	2.45 (0.756)	1.53 (0.198)		2.23 (1.030)	1.80 (0.296)		2.58 (1.040)	1.20 (0.249)	
−2 ≤ Z-score < 0	42.74 (2.481)	39.76 (0.799)		38.59 (3.975)	39.79 (1.070)		45.21 (3.033)	39.72 (1.092)	
0 ≤ Z-score < 2	51.39 (2.487)	54.46 (0.812)		54.51 (4.028)	53.90 (1.090)		49.54 (3.057)	55.15 (1.129)	
Z-score ≥ 2	3.42 (0.722)	4.25 (0.316)		4.66 (1.387)	4.51 (0.452)		2.68 (0.810)	3.93 (0.449)	
Weight Z-score	0.118 (0.064)	0.073 (0.020)	0.495	0.128 (0.112)	0.053 (0.027)	0.516	0.112 (0.075)	0.098 (0.028)	0.857
BMI Z-score	0.077 (0.068)	−0.056 (0.021)	0.061	0.048 (0.127)	−0.095 (0.028)	0.274	0.094 (0.075)	−0.009 (0.030)	0.200
**BMI percentile**			**0.169**			**0.534**			**0.349**
Underweight	7.12 (1.355)	9.14 (0.462)		10.48 (2.994)	10.69 (0.653)		5.12 (1.203)	7.23 (0.581)	
Normal	69.89 (2.221)	71.42 (0.689)		64.57 (4.079)	69.49 (0.959)		73.07 (2.513)	73.79 (1.015)	
Overweight	11.40 (1.481)	8.82 (0.418)		11.78 (2.362)	8.75 (0.580)		11.18 (1.921)	8.90 (0.620)	
Obesity	11.58 (1.502)	10.62 (0.501)		13.18 (2.840)	11.07 (0.678)		10.63 (1.711)	10.08 (0.736)	
**Subjective body image**			<**0.001**			**0.123**			<**0.001**
Lean	23.30 (2.196)	26.27 (0.701)		37.01 (4.190)	34.45 (1.019)		15.14 (2.189)	16.22 (0.830)	
Normal	35.83 (2.229)	43.26 (0.769)		29.57 (3.440)	37.66 (1.018)		39.56 (2.813)	50.13 (1.180)	
Obese	40.87 (2.426)	30.48 (0.715)		33.42 (4.052)	27.89 (0.888)		45.30 (2.968)	33.65 (1.124)	
Depressed mood	38.10 (2.45)	6.66 (0.40)	< 0.001	36.76 (3.99)	5.62 (0.50)	< 0.001	38.90 (2.87)	7.93 (0.65)	< 0.001
Suicide plan	27.29 (4.56)	0.00 (0.00)	< 0.001	21.86 (6.83)	0.00 (0.00)	< 0.001	31.08 (6.28)	0.00 (0.00)	< 0.001
Suicide attempt	11.75 (1.59)	0.01 (0.01)	< 0.001	11.96 (2.81)	0.00 (0.00)	< 0.001	11.62 (1.91)	0.01 (0.01)	< 0.001

Continuous variables are presented as means (standard error) and categorical data as percentages (standard error). BMI, body mass index.

### 3.3. Characteristics of the participants according to subjective body image and height Z-score

In the subgroup analysis, according to subjective body image, the proportions of depressed mood, suicide ideation, and suicide attempts were higher among all participants with perceived obesity than those with a normal body image (*p* = 0.027 for suicide attempts, all other *p* < 0.001) (Table 3). Among all participants with perceived obesity, 41.1% had a normal BMI. Among male participants with perceived obesity, 34.58% had a normal BMI.

**Table 3:** Comparison of subjects according to subjective body image.

	**Total**				**Male**				**Female**			
	**Lean**	**Normal**	**Obese**	***p***	**Lean**	**Normal**	**Obese**	***p***	**Lean**	**Normal**	**Obese**	***p***
Age, y	15.13 (0.06)	15.03 (0.04)	15.20 (0.05)	0.025	15.27 (0.07)	15.06 (0.06)	14.97 (0.07)	0.006	14.76 (0.10)	14.99 (0.06)	15.41 (0.07)	< 0.001
Height Z–score	0.17 (0.03)	0.20 (0.02)	0.28 (0.03)	0.013	0.15 (0.04)	0.23 (0.04)	0.33 (0.04)	0.005	0.20 (0.06)	0.17 (0.03)	0.23 (0.04)	0.423
Weight Z–score	−1.06 (0.02)	−0.10 (0.02)	1.25 (0.03)	< 0.001	−1.02 (0.03)	0.04 (0.03)	1.41 (0.04)	< 0.001	−1.16 (0.04)	−0.22 (0.02)	1.11 (0.04)	< 0.001
BMI Z–score	−1.38 (0.02)	−0.24 (0.01)	1.33 (0.03)	< 0.001	−1.34 (0.02)	−0.12 (0.02)	1.49 (0.04)	< 0.001	−1.47 (0.03)	−0.35 (0.02)	1.18 (0.04)	< 0.001
**BMI percentile**				<**0.001**				<**0.001**				<**0.001**
Underweight	32.25 (1.42)	1.31 (0.23)	0.05 (0.05)		30.19 (1.66)	0.60 (0.25)	0.00 (0.00)		37.34 (2.46)	1.92 (0.38)	0.09 (0.09)	
Normal	67.65 (1.42)	95.78 (0.42)	41.10 (1.33)		69.67 (1.66)	95.05 (0.69)	34.58 (1.80)		62.66 (2.46)	96.42 (0.50)	47.14 (1.80)	
Overweight	0.10 (0.08)	2.54 (0.32)	25.29 (1.12)		0.14 (0.11)	3.75 (0.59)	26.55 (1.68)		0.00 (0.00)	1.47 (0.32)	24.12 (1.46)	
Obesity	0.00 (0.00)	0.38 (0.13)	33.56 (1.33)		0.00 (0.00)	0.60 (0.26)	38.86 (1.91)		0.00 (0.00)	0.18 (0.09)	28.65 (1.72)	
Depressed mood	9.01 (0.84)	8.13 (0.60)	11.92 (0.89)	< 0.001	8.20 (1.00)	6.65 (0.82)	8.20 (1.05)	0.379	11.02 (1.61)	9.43 (0.89)	15.37 (1.39)	< 0.001
Suicide ideation	8.25 (0.89)	7.74 (0.57)	11.96 (0.84)	< 0.001	6.86 (0.97)	5.11 (0.67)	7.60 (1.09)	0.123	11.67 (1.73)	10.04 (0.88)	16.00 (1.28)	< 0.001
Suicide plan	1.04 (0.51)	0.86 (0.29)	1.79 (0.51)	0.261	0.94 (0.62)	0.43 (0.31)	0.98 (0.57)	0.662	1.27 (0.90)	1.22 (0.47)	2.49 (0.82)	0.324
Suicide attempt	2.16 (0.67)	1.14 (0.29)	2.90 (0.59)	0.027	2.07 (0.82)	0.94 (0.41)	1.55 (0.70)	0.429	2.36 (1.14)	1.30 (0.41)	3.94 (0.90)	0.020

Continuous variables are presented as means (standard error) and categorical data as percentages (standard error). BMI, body mass index.

**Figure 2:**
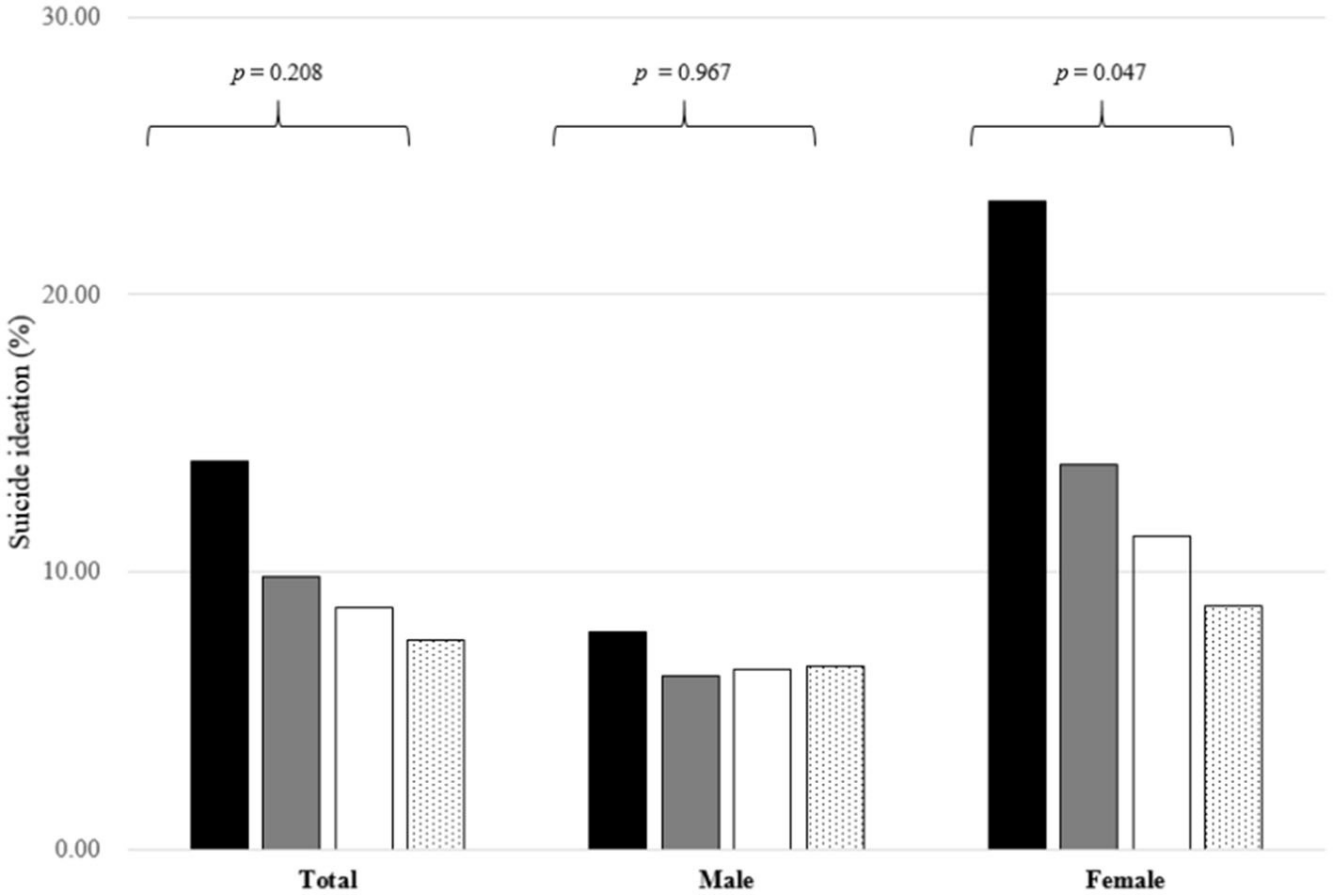
Proportions of individuals with suicide ideation according to height Z-score among the participants. The black bar represents individuals with a height Z-score below −2, the gray bar, with a height Z-score between −2 and 0, the white bar, with a height Z-score between 0 and 2, and the bar with the dotted pattern, with a height Z-score above 2. The number on the bar is the *p*-value of the analysis of variance.

The proportions of depressed mood, suicide ideation, and suicide attempts were higher among female participants with perceived obesity than those with a normal body image (*p* = 0.020 for suicide attempts, all other *p* < 0.001). Among female participants with perceived obesity, 47.17% had a normal BMI. In the subgroup analysis, according to the height Z-score, the proportion of female participants with suicide ideation decreased with an increase in the height Z-score (*p* = 0.047) (Figure 2).

### 3.4. Risk factors for suicide ideation in logistic regression analysis

On logistic regression, the odds ratios (ORs) (95% confidence intervals [CIs]) of height Z-score, perceived obesity, and depressed mood in relation to suicide ideation were 0.898 (0.818–0.986), 6.304 (5.903–7.803), and 6.304 (5.903–7.803), respectively, among all participants (Table 4). Among male participants, the ORs (95% CIs) of perceived obesity and depressed mood in relation to suicide ideation were 1.526 (1.019–2.284) and 6.590 (4.676–7.803), respectively. Among female participants, the ORs (95% CIs) of height Z-score, perceived obesity, and depressed mood were 0.874 (0.773–0.987), 5.685 (4.342–7.444), and 5.685 (4.342–7.444), respectively. In addition, female participants with height Z-scores of above 2 were less likely to express suicide ideation than those with height Z-scores below −2 (OR = 0.317, 95% CI = 0.104–0.965).

**Table 4:** Logistic regression for suicide ideation.

	**Total**		**Male**		**Female**	
	**OR (95% CI)**	***p***	**OR (95% CI)**	***p***	**OR (95% CI)**	***p***
Age, y	1.030 (0.982–1.079)	0.225	1.044 (0.969–1.126)	0.257	1.021 (0.960–1.087)	0.506
Height Z-score	0.898 (0.818–0.986)	0.024	0.943 (0.825–1.079)	0.392	0.874 (0.773–0.987)	0.030
Height group						
Z-score < -2	ref		ref		ref	
−2 ≤ Z-score < 0	0.671 (0.339–1.326)	0.251	0.780 (0.287–2.119)	0.626	0.529 (0.210–1.328)	0.175
0 ≤ Z-score < 2	0.589 (0.298–1.163)	0.127	0.813 (0.300–2.207)	0.685	0.417 (0.1660–1.050)	0.063
Z-score ≥ 2	0.502 (0.226–1.117)	0.091	0.832 (0.264–2.625)	0.854	0.317 (0.104–0.965)	0.043
Weight Z-score	1.029 (0.948–1.117)	0.492	1.045 (0.916–1.193)	0.512	1.010 (0.905–1.128)	0.857
BMI Z-score	1.079 (0.998–1.165)	0.055	1.078 (0.946–1.228)	0.261	1.067 (0.968–1.177)	0.193
BMI percentile						
Underweight	0.797 (0.525–1.209)	0.286	1.055 (0.548–2.032)	0.873	0.717 (0.429–1.198)	0.204
Normal	ref		ref		ref	
Overweight	1.322 (0.972–1.797)	0.075	1.449 (0.893–2.350)	0.133	1.268 (0.839–1.916)	0.260
Obesity	1.114 (0.818–1.516)	0.493	1.281 (0.772–2.126)	0.337	1.065 (0.723–1.568)	0.749
Subjective body image						
Lean	1.071(0.817–1.403)	0.619	1.368 (0.923–2.027)	0.119	1.183(0.812–1.724)	0.381
Normal	ref		ref		ref	
Obese	1.619 (1.294–2.026)	< 0.001	1.526 (1.019–2.284)	0.040	1.706 (1.305–2.230)	< 0.001
Depressed mood	6.304 (5.903–7.803)	< 0.001	6.590 (4.676–9.288)	< 0.001	5.685 (4.342–7.444)	< 0.001

OR, odds ratio; CI, confidence interval; BMI, body mass index.

**Figure 3:**
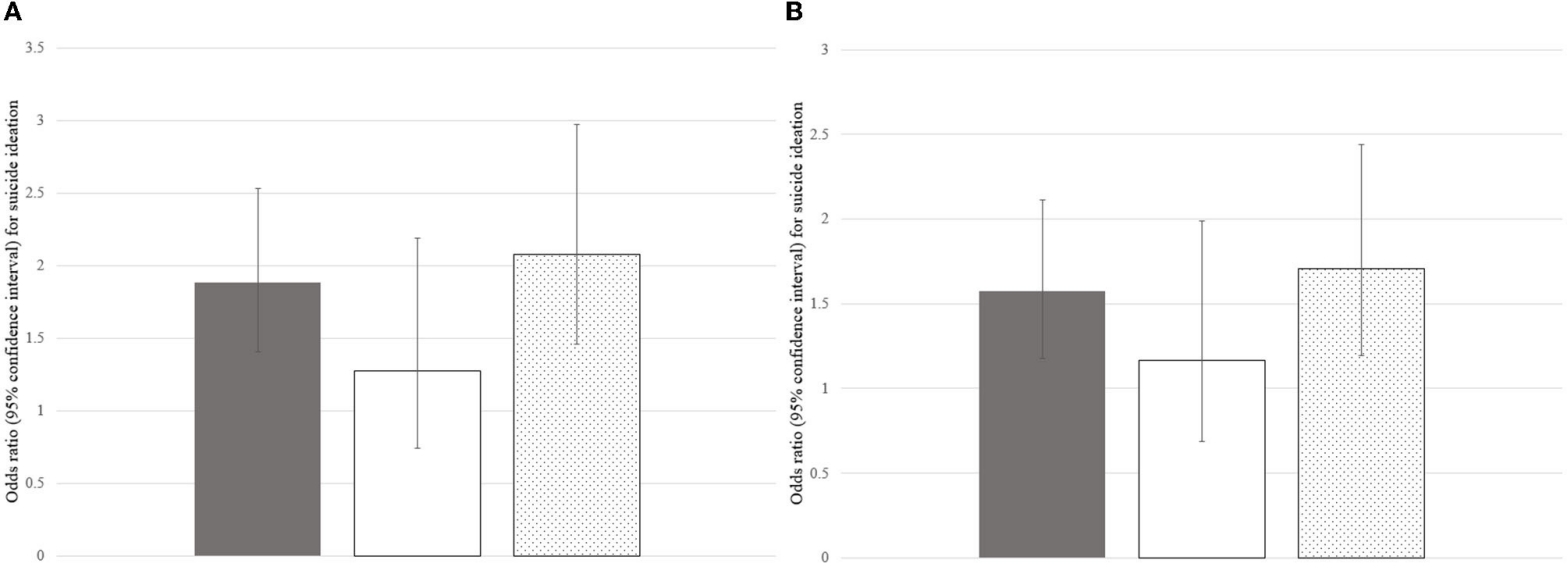
ORs (95% CIs) of perceived obesity for suicide ideation after adjusting for confounding factors. **(A)** ORs (95% CIs) of perceived obesity for suicide ideation after adjusting for age, height Z-score, and weight Z-score. **(B)** ORs (95% CIs) of perceived obesity for suicide ideation after adjusting for age, height Z-score, weight Z-score, and depressed mood. The gray, white, and dotted bars represent the ORs (95% CIs) of perceived obesity for suicide ideation among all participants, male participants, and female participants, respectively. OR, odds ratio; CI, confidence interval.

After adjusting for age, height Z-score, and weight Z-score, the ORs (95% CIs) of perceived obesity for suicide ideation were 1.886 (1.699–2.528) for all participants, 1.277 (0.745–2.189) for male participants, and 2.080 (1.456–2.971) for female participants (*p* = 0.375 for male participants, all other *p* < 0.001) (Figure 3A). After adjusting age, height Z-score, weight Z-score, and depressed mood, the ORs (95% CIs) of perceived obesity for suicide ideation were 1.575 (1.175–2.111) for all participants, 1.168 (0.686–1.989) for male participants, and 1.706 (1.192–2.441) for female participants (*p* = 0.002 for the total number of participants, *p* = 0.568 for male participants, and *p* = 0.003 for female participants) (Figure 3B).

**Figure 4:**
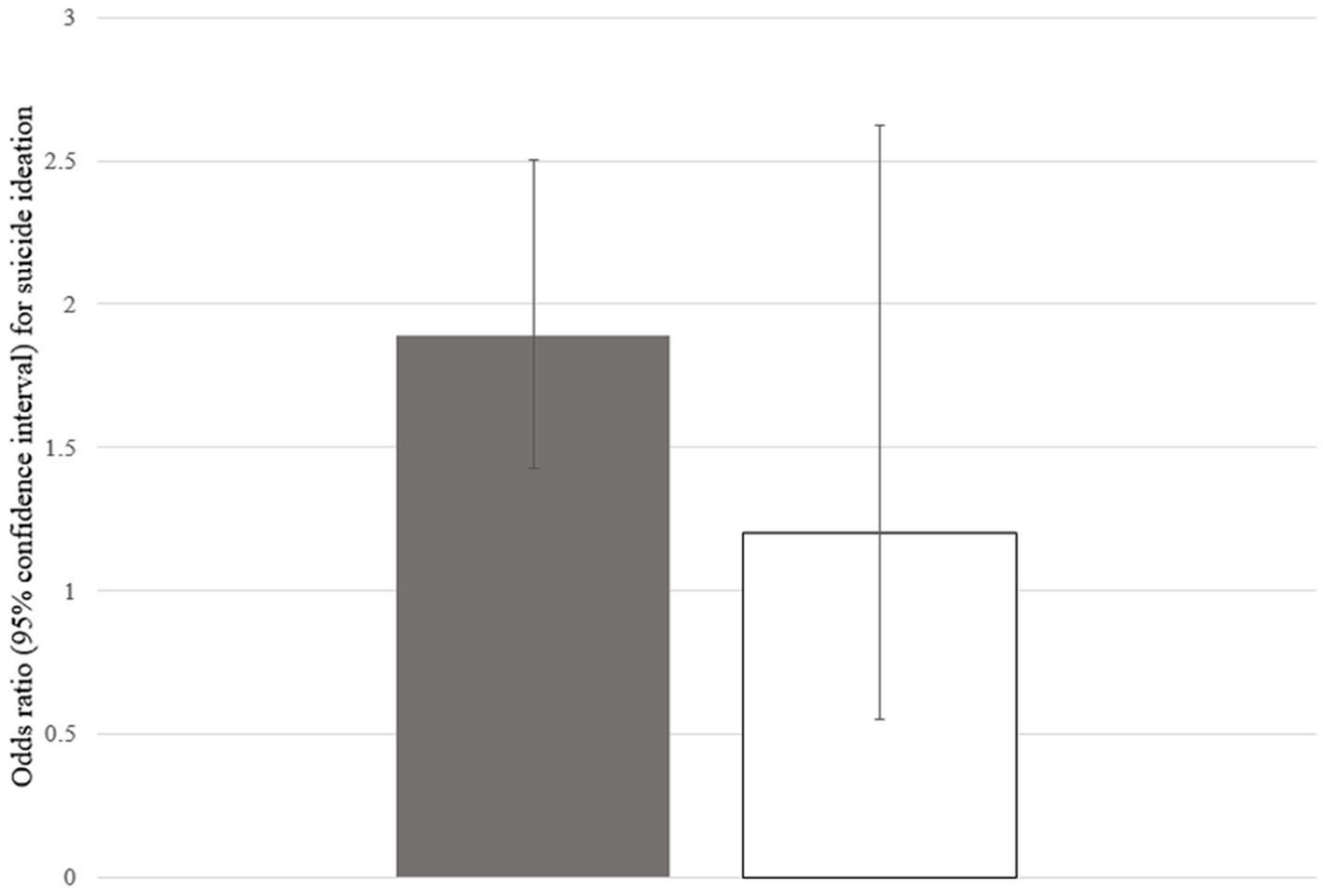
ORs (95% CIs) of perceived obesity for suicide ideation according to BMI. The gray bar represents ORs (95% CIs) of perceived obesity for suicide ideation among the participants with normal BMI or underweight and the white bar among the participants with overweight or obesity. OR, odds ratio; CI, confidence interval; BMI, body mass index.

In the subgroup analysis, according to BMI, the ORs (95% CIs) of perceived obesity for suicide ideation were 1.889 (1.426–2.501) for the participants with normal BMI or underweight and 1.202 (0.551–2.622) for the participants who were overweight or obese (Figure 4).

In the subgroup analysis, according to the KNHANES phase, the OR (95% CI) of height Z-score for suicide ideation was 0.757 (0.582–0.985) for KNHANES VI (Supplementary Table 1). The ORs (95% CIs) of subjective body image for suicide ideation were significant for KNHANES IV, V, and VI, and the ORs (95% CIs) of depressed mood for suicide ideation were significant for KNHANES IV, V, VI, and VII. Interaction p-values were not significant for variables.

## 4. Discussion

This study demonstrated the following: (1) perceived obesity was positively associated with the proportion of suicide ideation in adolescents, even among those who fell within the normal BMI range or were underweight; (2) shorter height, even if not of short stature, was associated with suicide ideation in adolescents. These relationships were more prominent among girls than boys. Additionally, a substantial proportion of the participants perceived themselves as being obese even though they were not technically obese or overweight. The proportion of perceived obesity was higher among girls than boys, although the proportion of actual obesity was lower among girls.

In our study, suicide ideation was associated with perceived obesity rather than actual obesity or BMI Z-score, possibly because of weight stigma. Interestingly, research suggests that the perceptions of body weight in adolescents are more important than actual body weight (23, 34). In this study, perceived obesity was positively associated with suicide ideation after adjusting for age, height Z-score, body weight Z-score, and depressed mood. Importantly, weight stigma is not restricted only to obese adolescents. Indeed, Puhl et al. (35) reported that 19% of US adults of normal weight perceived themselves as being overweight. Cruz-Sáez et al. (36) reported that 12.3% of normal-weight women had extreme weight-control behaviors.

We noted that a relatively short height was negatively associated with suicide ideation among adolescents even though they were not particularly short. Considering previous reports suggesting that short stature has no relationship with psychosocial burden (37), we think it would be controversial to insist that short stature induces suicide ideation or vice versa. Nevertheless, short stature in childhood and adolescence can lead to life dissatisfaction and poor subjective health experiences (38). The association between short stature and suicide ideation may be more psychological in nature, such as due to negative self-image, than endocrinological.

As reflected in our results, the association between suicide ideation and perceived obesity or short stature seems to be related to negative body image, although the causality could not be explained. Negative body image should be considered both in psychological and environmental aspects. First, negative body image is related to a distorted perception of body image and is associated with low self-esteem, which can lead adolescents to have a negative self-image (23, 39). A negative self-image can be a predictor of not only psychological problems but also emotional distress (40, 41). Emotional distress is known to be linked to diverse negative cognitive patterns, such as selective processing of depressive information, making dysfunctional attributions, and engaging in negative automatic thinking (42). Moreover, adolescents, especially girls, with major depression tend to have a poorer self-image (43, 44). Considering that body image might be established in early adolescence, careful monitoring of emotional distress in children and adolescents is necessary to prevent negative outcomes as a result of negative body image (45, 46). Second, the influence of media tends to encourage a distorted body image since slim characteristics are often portrayed as being popular and attractive, whereas obese characteristics are depicted as being unpopular and in a negative light (24). In this regard, the association between negative body image and suicide ideation can be explained by the interpersonal theory of suicide, namely, perceived burdensomeness and thwarted belongingness are predictors of the desire to engage in suicide (47). Hunger et al. (25) reported that perceived burdensomeness can mediate weight stigma, and suicide ideation and negative weight stigma could be more powerful risk factors for suicide than BMI itself. Third, especially in adolescence, the association between weight stigma and psychological distress can be stronger, owing to teasing by peers (24, 48). Weight-based teasing is widespread and one of the risk factors for body dissatisfaction (48–50). A population-based study reported that weight-based harassment was associated with low self-esteem, depression, and body dissatisfaction among girls (51). A US study with a nationally representative survey reported that perceived obesity was positively related to suicide ideation, planning, and attempts among adolescents (48).

In our study, the proportion of participants exhibiting suicide ideation and depressed mood was higher among girls than boys; the relationship between perceived obesity and psychological stress was also more prominent among girls than boys. These findings may be attributed to gender differences in emotional expression and the fact that the burden of weight stigma is greater for female participants than for male participants with regard to attractiveness. A KNHANES-based study reported that women with an overweight body image had a higher level of depressive symptoms than those without; this finding was not observed in men (52). Carpenter et al. (20) reported that BMI was negatively related to suicide ideation and attempts in men, whereas it was positively related to both in women. A school based-study reported that obesity was related to psychological distress among girls but not among boys (53).

This study has some limitations. First, this was a cross-sectional study limited to South Korea, which limits the generalizability and causality of our results. In addition, the onset age of suicidal ideation and obesity was not specified in this study. Second, we could not consider confounding factors such as puberty, teasing, and sexual orientation because this information was not provided in the KNHANES. Third, environmental factors including familial factors were not considered in this study. Fourth, it was impossible to conduct structured interviews or use measurement instruments, as this study was based on a large-scale adolescent survey. Self-reported items cannot entirely represent an adolescent's mental health, and our study did not include every psychiatric disorder. To evaluate the effect of psychiatric disorders precisely, structured interviews such as the Structured Clinical Interview for the Diagnostic and Statistical Manual of Mental Disorders (DSM) should be considered (54). Psychometric instruments such as the Beck depression inventory for depression (55) and the Columbia-suicide severity rating scale for suicidal ideation could be useful for evaluating psychiatric disorders (56). Fifth, we considered only diabetes, congenital heart disease, and attention deficit hyperactivity disorder among chronic diseases associated with psychological distress. Sixth, we could not assess the effect of treatment for depression and suicide ideation because the KNAHNES provides information on medical treatment for psychiatric disease, although pharmacotherapy including antidepressants such as buprenorphine is the recommended treatment strategy for depression (57). However, the study assessed the association of suicide ideation with BMI, height, and subjective body image using nationally representative data from a large number of adolescents.

## 5. Conclusion

Our study demonstrated that perceived obesity was associated with suicide ideation among adolescents even though they were not really obese. In addition, a relatively short stature was associated with suicide ideation among adolescents even though they were not really short. These associations were more apparent among girls than boys. The findings emphasize a mutual relationship between psychological distress, negative body image, and height and subjective body image in adolescents. In addition, our results emphasize the importance of assessing the risk of suicide ideation in adolescents with perceived obesity and those with relatively short stature. Moreover, adolescents, especially girls, with psychological problems due to body image dissatisfaction, need attentive care from physicians and family members even though they may not be obese. Therefore, it is necessary to determine subjective feelings and psychological distress during the assessment of obesity and growth in adolescents to prevent suicide ideation.

## Data availability statement

The raw data supporting the conclusions of this article will be made available by the authors, without undue reservation.

## Ethics statement

The studies involving human participants were reviewed and approved by the Institutional Review Board of the Yonsei University Gangnam Severance Hospital. Written informed consent to participate in this study was provided by the participants' legal guardian/next of kin.

## Author contributions

KS, JL, SL, and HC: conceptualization. KS, JL, and HC: design, methodology, and conducted the study. KS, JL, SJ, and HL: statistical analysis and interpretation. KS and JL: writing—original draft preparation. HC: writing—reviewing and editing. KS: resources. H-SK: supervision. All authors contributed to the article and approved the submitted version.
