# Supplementary material for: Height and subjective body image are associated with suicide ideation among Korean adolescents
Source: Front Psychiatry. 2023 Jun 12;14:1172940. doi: 10.3389/fpsyt.2023.1172940 (PMC10291136; doi:10.3389/fpsyt.2023.1172940)
Supplement: Supplementary file 1 [file Table_1.docx]

Supplementary Material

Height and Subjective Body Image are Associated with Suicide Ideation Among Korean Adolescents

**Kyungchul Song^1†^, Junghan Lee^2†^, San Lee^2^, Soyoung Jeon^3^, Hye Sun Lee^3^, Ho-Seong Kim^1^, Hyun Wook Chae^1*^**

^†^Kyungchul Song and Junghan Lee contributed equally as co-first authors.

^1^Department of Pediatrics, Yonsei University College of Medicine, Seoul 03722, Republic of Korea

^2^Department of Psychiatry, Institute of Behavioral Sciences in Medicine, Yonsei University College of Medicine, Seoul 03722, Republic of Korea

^3^Biostatistics Collaboration Unit, Yonsei University College of Medicine, Seoul 03722, Republic of Korea

**Correspondence:**

Hyun Wook Chae

[hopechae@yuhs.ac](mailto:hopechae@yuhs.ac)

# Supplementary Table

**
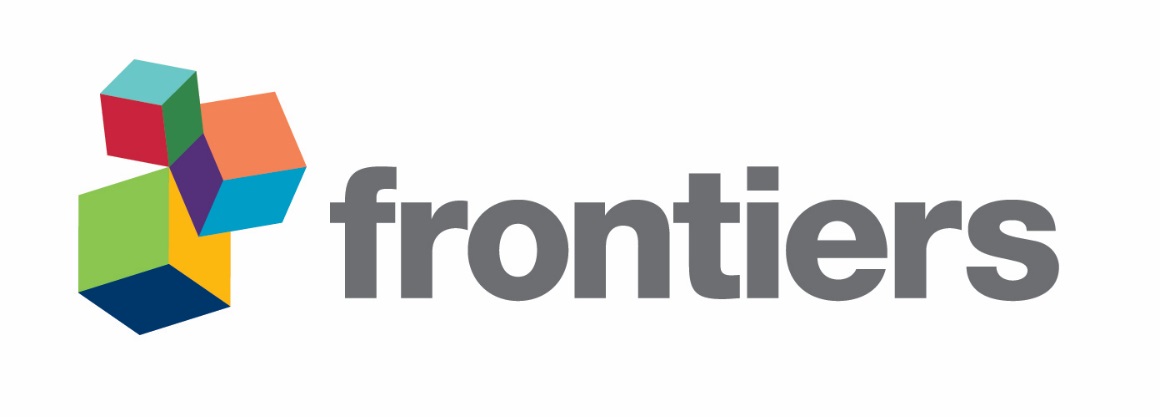
**

**Supplementary Table 1.** Logistic regression for suicide ideation according to KNHANES phase

|  | Ⅳ (2007–2009) | | Ⅴ (2010–2012) | | Ⅵ (2013–2015) | | Ⅶ (2016–2018) | | Interaction p-value |
| --- | --- | --- | --- | --- | --- | --- | --- | --- | --- |
|  | OR (95% CI) | *p* | OR (95% CI) | *p* | OR (95% CI) | *p* | OR (95% CI) | *p* |  |
| Age, y | 0.972 (0.899-1.050) | 0.473 | 1.078 (0.998-1.164) | 0.058 | 1.058 (0.924-1.211) | 0.416 | 1.210 (1.042-1.405) | 0.013 | 0.054 |
| Height Z-score | 0.906 (0.802-1.024) | 0.114 | 0.937 (0.793-1.107) | 0.444 | 0.757 (0.582-0.985) | 0.038 | 0.981 (0.710-1.356) | 0.908 | 0.537 |
| Height group |  |  |  |  |  |  |  |  |  |
| Z-score <-2 | ref |  | ref |  | ref |  | ref |  | 0.603 |
| -2 ≤ Z-score < 0 | 1.028 (0.411-2.572) | 0.952 | 1.350 (0.377-4.839) | 0.645 | 0.152 (0.031-0.751) | 0.021 | 0.740 (0.097-5.622) | 0.771 |  |
| 0 ≤ Z-score < 2 | 0.779 (0.313-1.938) | 0.591 | 1.283 (0.356-4.629) | 0.703 | 0.139 (0.029-0.670) | 0.014 | 0.633 (0.084-4.754) | 0.657 |  |
| Z-score ≥ 2 | 0.925 (0.309-2.767) | 0.889 | 0.815 (0.176-3.778) | 0.794 | 0.132 (0.021-0.849) | 0.033 | 0.281 (0.017-4.565) | 0.372 |  |
| Weight Z-score | 1.094 (0.964-1.241) | 0.165 | 1.066 (0.926-1.226) | 0.372 | 0.973 (0.754-1.255) | 0.834 | 1.004 (0.760-1.328) | 0.976 | 0.846 |
| BMI Z-score | 1.151 (1.021-1.299) | 0.022 | 1.101 (0.953-1.273) | 0.191 | 1.091 (0.909-1.311) | 0.349 | 1.016 (0.774-1.334) | 0.909 | 0.851 |
| BMI percentile |  |  |  |  |  |  |  |  | 0.536 |
| Underweight | 0.636 (0.304-1.331) | 0.23 | 0.593 (0.302-1.165) | 0.129 | 1.437 (0.534-3.866) | 0.473 | 1.345 (0.453-3.993) | 0.593 |  |
| Normal | ref |  | ref |  | ref |  | ref |  |  |
| Overweight | 1.082 (0.666-1.760) | 0.749 | 1.887 (1.172-3.037) | 0.009 | 0.889 (0.382-2.069) | 0.785 | 0.903 (0.203-4.022) | 0.893 |  |
| Obesity | 1.096 (0.654-1.835) | 0.729 | 1.212 (0.687-2.136) | 0.507 | 1.411 (0.710-2.804) | 0.326 | 1.266 (0.520-3.085) | 0.603 |  |
| Subjective body image |  |  |  |  |  |  |  |  | 0.216 |
| Lean | 0.780 (0.508-1.198) | 0.257 | 1.085 (0.681-1.731) | 0.73 | 1.985 (0.949-4.149) | 0.068 | 1.315 (0.586-2.954) | 0.507 |  |
| Normal | ref |  | ref |  | ref |  | ref |  |  |
| Obese | 1.714 (1.221-2.406) | 0.002 | 1.763 (1.202-2.588) | 0.004 | 2.230 (1.155-4.308) | 0.017 | 0.980 (0.426-2.253) | 0.962 |  |
| Depressed mood | 7.800 (5.537-10.986) | <0.001 | 7.648 (4.850-12.062) | <0.001 | 10.304 (5.659-18.763) | <0.001 | 16.307 (8.229-32.316) | <0.001 | 0.238 |

KNHANES, Korea National Health and Nutrition Examination Survey; OR, odds ratio; CI, confidence interval; BMI, body mass index.
